# Immunoglobulin genes and diversity: what we have learned from domestic animals

**DOI:** 10.1186/2049-1891-3-18

**Published:** 2012-06-20

**Authors:** Yi Sun, Zhancai Liu, Liming Ren, Zhiguo Wei, Ping Wang, Ning Li, Yaofeng Zhao

**Affiliations:** 1 State Key Laboratory of Agrobiotechnology, College of Biological Sciences; National Engineering Laboratory for Animal Breeding, China Agricultural University, Beijing, 100193, P. R. China; 2 Department of Biochemistry, Jiaozuo Teachers Colleague, Jiaozuo, 454001, Henan, P. R. China; 3 College of Animal Science and Technology, Henan University of Science and Technology, Henan, 471003, P. R. China

**Keywords:** Diversity, Domestic animals, Immunoglobulin gene

## Abstract

This review focuses on the diversity of immunoglobulin (Ig) genes and Ig isotypes that are expressed in domestic animals. Four livestock species—cattle, sheep, pigs, and horses—express a full range of Ig heavy chains (IgHs), including μ, δ, γ, ϵ, and α. Two poultry species (chickens and ducks) express three IgH isotypes, μ, υ, and α, but not δ. The κ and λ light chains are both utilized in the four livestock species, but only the λ chain is expressed in poultry. V(D)J recombination, somatic hypermutation (SHM), and gene conversion (GC) are three distinct mechanisms by which immunoglobulin variable region diversity is generated. Different domestic animals may use distinct means to diversify rearranged variable regions of Ig genes.

## Introduction

Immunoglobulins (Igs) are essential molecules for the animal adaptive immune response and are expressed only in jawed vertebrates, including fish, amphibians, reptiles, birds, and mammals. These molecules are usually expressed in either a membrane-bound form (B cell receptor) or a secreted form (antibody). Regardless of the form, a typical immunoglobulin molecule contains a heterodimer consisting of two identical heavy chains and two identical light chains. In a very small number of species, such as sharks and camels, antibodies consisting only of heavy chains have been identified [1,2]. Both heavy (H) and light (L) chains contain functionally distinct variable (V) and constant (C) regions, and variable regions from both the heavy and light chains form the antigen binding sites. The C regions of the two heavy chains, which determine the isotypes of the antibody, are responsible for effector functions through their interactions with a variety of receptors expressed on immune cells. Throughout the evolution of immunoglobulin genes in jawed vertebrates, key questions addressed by researchers are how various mechanisms generate the immunoglobulin V region repertoire and how C regions are diversified to generate different isotypes with distinct effector functions. Comparative studies have revealed three distinct mechanisms by which immunoglobulin V region diversity is generated. One is V(D)J recombination, a common mechanism that is utilized by most animals to generate V diversity through somatic DNA rearrangement, which is a key feature of animal adaptive immunity. In most vertebrates, there are as many as hundreds of variable gene segments and a number of diversity (D) and joining (J) segments in the Ig gene locus (in the case of the Ig light chain gene locus, there are no D segments). Somatic V(D)J rearrangements are able to provide thousands of V(D)J combinatorial sets. Nonetheless, V(D)J recombination appears to be an imprecise process that is often accompanied by nucleotide insertions or deletions at the VD and DJ junction sites, although it is a site-specific process mediated by recombination signal sequences (RSSs) flanking the V (at the 3’ end), D (at both 5’ and 3’ ends), and J (at the 5’ end) genes. Through this imprecise recombination, a given set of V, D, and J can be used to generate hundreds of different junction sequences, although only one-third of these sequences are in the correct frame for translation [3]. Another mechanism is somatic hypermutation (SHM), which occurs in all jawed vertebrates and can intentionally introduce non-template mutations into the variable regions of the transcribed Ig genes. SHM occurs at a rate of approximately 10^-3^ mutations per base pair per cell division, which is 10^6^-fold higher than the spontaneous mutation rate in somatic cells [4]. A third mechanism is gene conversion (GC), a process that utilizes pseudo V genes to non-reciprocally modify a pre-rearranged V gene repeatedly [5]. Finally, as both IgH and IgL chains can be diversified by the above mechanisms, the nature of VH and VL pairing of antibodies further multiplies the antigen binding repertoire [6].

Over the past few decades, Ig genes in mice and humans have been intensively studied, and these efforts have contributed immensely to our understanding of the generation of antibody diversity and other relevant molecular mechanisms. However, a significant lesson from comparative studies has also taught us that different animals may utilize different mechanisms to accomplish the same aim. In this regard, we have also learned a great deal by studying Ig genes from other species, including domestic animals.

## Cattle and sheep Ig genes

Both cattle and sheep belong to the order *Artiodactyla*, suborder *Ruminantia*, and family *Bovidae*. Ig genes from these two species are very similar in many aspects. Below, we focus on cattle to describe the major findings related to their Ig genes.

Like humans and mice, cattle express five classes of IgH chains: μ, δ, γ, ϵ, and α. Compared with the presence of four subclasses of IgG (encoded by γ1, γ2, γ3, and γ4) and two subclasses of IgA (encoded by α1 and α2) in humans, only three γ genes (γ1, γ2, and γ3) and a single α gene have been identified in cattle [7,8]. The existence of a bovine δ gene was not confirmed until 2002, when we cloned the bovine δ gene using an EST (expressed sequence tag)-based approach [9]. It was thought that the δ gene might be missing in cattle because of failed attempts to clone the gene or identify the presence of IgD protein. Unlike its counterpart in humans and mice, the bovine δ gene (as well as the porcine and ovine δ genes) contains a μ-like CH1 exon and a short switch region δ (Sδ) sequence, both of which were originally acquired through a duplication of μCH1 and Sμ [9]. The presence of the Sδ sequence allows the bovine δ gene to be expressed by class switch recombination (CSR) (our unpublished data).

It is particularly interesting that cattle (as well as sheep) appear to have two IgH loci in their genomes: one in BTA21 and one in BTA11 [10,11]. The IgH locus in BTA21 has been shown to contain all functional IgH isotype-encoding genes, including μ, δ, γ, ϵ, and α [10,12]. Although it was originally believed that the IgH locus in BTA11 only contained a pseudo μ gene, our recent sequencing of a BAC clone covering this region showed that a functional μ gene and a pseudo δ gene exist in this region [11] and our unpublished data]. Both μ genes (in BTA21 and BTA11) are transcribed and are functional, but the latter is typically transcribed at a very low level (our unpublished data). It would be very interesting to investigate how these two loci interact during an immune response and during the development of bovine B cells because in many transgenic mouse models, the presence of an additional IgH locus usually inhibits the expression of endogenous IgH genes and disturbs normal B cell development [13-15].

Compared with humans and mice, cattle have a very limited number of germline heavy chain V, D, and J segments. Only a single VH family, designated as BoVH1, is expressed at the cDNA level, and it is thought that the bovine genome contains no more than 20 VH segments [16,17]. Only nine DH and four functional JH segments (two in BTA21 locus and another two in BTA11) have been identified. Nonetheless, the bovine DH segments can be exceptionally lengthy. One bovine DH (termed DH2) consists of 149 nucleotides, accounting for 49 amino acid codons and representing perhaps the longest DH in any of the jawed vertebrates examined to date. This long DH contributes significantly to the length of the complementarity-determining region 3 (CDR3) of the bovine heavy chain, which may extend up to 61 amino acids [7,18-20]. As the CDR3 length in most species is approximately 10 amino acids on average, this exceptionally long CDR3 is quite unusual and remarkable. It would be very interesting to address the immunological significance of this long CDR3.

Although similar to humans and mice, two types of Ig light chains (λ and κ) are expressed in cattle, though the λ/κ ratio differs significantly among these animals. In mice, approximately 96% of light chains in the serum are the κ type, while the κ type in humans accounts for only 66% of the total population of Ig light chains. In contrast, the light chain repertoire in cattle is dominated by λ chains (96%) [6]. To some extent, the λ/κ ratio at the protein level in different species is thought to be a reflection of the chain that has the greatest number of V genes in the genome [21]. This appears to be true in cattle as well, as a recent analysis based on the bovine genome identified 25 functional Vλ but only eight functional Vκ genes [22].

## Pig Ig genes

Pigs belong to the order *Artiodactyla*, suborder *Suina*, and family *Suidae*. Pig species also express five IgH isotypes (μ, δ, γ, ϵ, and α), in which the μ, δ, ϵ, and α genes are all present in a single copy at the IgH locus. In contrast, the porcine γ gene is extensively diversified and thus can develop multiple subclass-encoding genes; at least six distinct γ genes and some allelic variants have been defined in pigs [23,24]. The porcine δ gene shares a similar feature with its counterparts in cattle and sheep because it also has a μ-like CH1 and an Sδ [9]. Despite not having been demonstrated, it is also expected that the porcine δ gene can be expressed through CSR mediated by the Sδ region.

Mapping of the porcine IgH locus revealed only two functional DH and a single functional JH, suggesting that V(D)J recombination contributes little to the VH repertoire in pigs [24]. Previous studies have suggested that all expressed porcine VH genes belong to a single family corresponding to human VH3. A partial characterization of the VH locus has identified 15 VH genes, of which only 10 are structurally functional [25,26].

Unlike other species, pigs have a balanced λ/κ ratio (~52% vs. 48%) at the protein level [27]. Consistent with the rule mentioned above, similar numbers of functional Vλ and Vκ genes have been observed at both light chain loci [28]. Mapping of the porcine λ locus indicates the presence of three pairs of Jλ-Cλ. Upstream of the Jλ-Cλ pairs, 22 Vλ genes were identified, of which only nine appeared to be functional. As for the κ locus, there is a single Cκ preceded by five Jκ segments. Although 14 Vκ genes could be identified, only nine of these are functional, resulting in the same number of functional Vλ genes [29].

## Horse Ig genes

Horses belong to the order *Perissodactyla*, suborder *Equidae*, and family *Equus*. Similar to all artiodactyls, horses also have single copies of the μ, δ, ϵ, and α genes and a large number of γ genes per haploid genome. The genomic organization of the horse IgH locus has been completely mapped, giving the order 5’-VH-DH-JH-μ-δ-γ1-γ2-γ3-γ7-γ4-γ6-γ5-ϵ-α-3’ [29]. The genomic gene structure and coding nucleotide sequence of the horse δ gene shares a higher degree of homology with human than artiodactyls δ genes, and there is no short Sδ located upstream of the horse δ gene [29]. Phylogenetic analysis of the horse γ genes indicated that they evolved by gene duplication and GC (or unequal crossing) after species separation, and the highest nucleotide sequences homology (96%) between the γ4 and γ7 genes suggested that these two γ genes duplicated most recently at the horse IgH locus [30,31].

Based on horse genomic data, 50 VH, 40 DH, and 8 JH segments were identified upstream of the CH locus. All VH segments can be classified into seven subgroups, which are distributed into all three mammalian VH clans. The VH segments from subgroup 2, corresponding to the BoVH1, are predominantly expressed at the cDNA level; however, the other VH subgroups are rarely expressed [32,33]. Because only four potentially functional germline VH gene segments have been characterized in subgroup 2 thus far, the recombinational diversity of the horse heavy chain locus appears to mainly depend on junctional flexibility, with 35 of 40 DH segments utilized in the expressed IgH cDNA. Additionally, extensive SHM was found in the variable regions of the transcribed IgH and IgL genes [31].

Similar to cattle and sheep, horses are another λ-predominant mammalian species [26]. The preference for the λ chain in horse Igs supports the above explanation that the germline V repertoire of the λ locus is larger than that of the κ locus. According to the horse genome sequence, the germline Vλ repertoire contains as many as 11 subgroups, which consist of at least 27 potentially functional Vλ segments, 5 ORFs, and 112 pseudogene segments. Among the Vλ segments, 110 were arranged upstream of the seven Jλ-Cλ clusters with the same transcriptional polarity as Jλ-Cλ; however, the remaining 34 Vλ segments localized downstream of the Jλ-Cλ clusters showed the opposite transcriptional polarity. The horse κ locus contains only a single Cκ gene, 5 Jκ genes, and 60 Vκ genes, of which the numbers of potentially functional genes, ORFs, and pseudogenes are 19, 2, and 39, respectively [31]. Similar to the heavy chain, several Vλ (or Vκ) segments from a single Vλ (or Vκ) subgroup were preferentially used in the expressed λ (or κ) chain repertoire [31,34,35]. Compared with the κ chain, the flexibility of the V-J junction in λ chain cDNA sequences is higher due to the large diversity of the λ CDR3 regions, particularly because of the addition of the non-templated (N) and palindromic (P) nucleotides [31].

## Chicken and duck Ig genes

Both chickens and ducks express only three classes of IgH chains, namely IgM, IgY (encoded by υ gene), and IgA. Although the IgD-encoding gene has now been identified in nearly all groups of jawed vertebrates, no bird species has been found that contains an IgD-encoding gene [36,37]. As the most conserved IgH class, the IgM in birds has a very similar structure to the mammalian IgM [38-40].

Bird IgY is functionally equivalent but structurally different from mammalian IgG. IgY consists of four CH domains but contains no hinge region [41]. Interestingly, although both chickens and ducks express the intact 4-CH IgY form, a truncated IgY form lacking the last two CH domains, designated as IgY(ΔFc), is also expressed in ducks. Duck IgY(ΔFc) heavy chain is transcribed from the same υ gene as the intact IgY but with different transcriptional termination sites [42]. Structurally, IgY(ΔFc) maintains antigen-binding activity but lacks effector function due to the absence of the Fc region. The immunological advantages of this IgY form remain unknown; nevertheless, this is a very interesting question that must be addressed.

Despite being orthologous to mammalian IgA, bird IgA differs structurally from its mammalian counterpart because it has four CH domains and no hinge region [38,39,43]. The mammalian α gene is always located in the most 3’ region of the IgH locus. In both chickens and ducks, however, the α gene is positioned in the middle of the IgH locus and is inverted, which is a different transcriptional orientation from that of μ and υ [35,36]. Despite this, our unpublished data shows that the bird α gene is still expressed through a CSR process.

The most interesting observation obtained from studies of chicken Ig genes is the mechanism used by this species to generate immunoglobulin diversity. In the chicken IgH locus, only a single functional VH, 16 DH, and a single JH segment have been identified [44,45]. Therefore, V(D)J recombination in chickens can provide only very limited diversity to the IgH chains. However, there are approximately 80–100 pseudo VH genes upstream of the single VH [43]. Analyses of expressed chicken VH segments revealed that instead of V(D)J recombination, GC plays a major role in the generation of VH diversity in chickens. As a nonreciprocal process, GC can use the upstream pseudo VH segments as donor sequences to repeatedly modify the functional VH that has been recombined with D and J segments. This mechanism ensures that all expressed chicken VH have a single leader sequence (from the single functional VH) and largely diverse coding regions [5,43].

**Figure 1:**
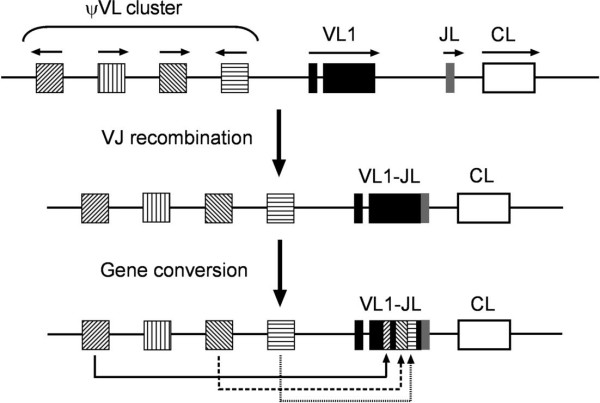
**V-J recombination and gene conversion in the chicken light chain locus.** The transcription orientation of each gene segment is indicated by an arrow.

Two types of IgL chains (λ and κ) are found in both mammals and reptiles, two groups of animals that are phylogenetically close to birds. It is quite surprising that only the λ chains are expressed in both chickens and ducks [46,47]. In the chicken λ locus, similar to the heavy chain, there is only a single Vλ and more than 20 pseudo Vλ present upstream of a single pair of Jλ-Cλ. Accordingly, GC, instead of VJ recombination, is the major mechanism responsible for IgL diversity (Figure 1) [5,46,48].

## Conclusion

Studies that have analyzed the Ig genes in domestic animals have generated novel information that we could not learn from research solely focusing on humans and mice, thereby indicating the importance of comparative studies. This new information also emphasizes the fact that different species may use distinct mechanisms to fulfill a principally similar function. Compared with humans and mice, it appears that all of the domestic animals described above have a relatively smaller germline V gene repertoire. Future studies are needed to completely characterize Ig gene loci and the mechanism responsible for diversity generation in these species. A better understanding of the genetic components of the animal immune system, including the Ig genes, would surely be useful in the study of disease control in farm animals.

## Abbreviations

Ig: Immunoglobulin; H: Heavy; L: Light; V: Variable; D: Diversity; J: Joining; C: Constant; RSSs: Recombination signal sequences; SHM: Somatic hypermutation; GC: Gene conversion; EST: Expressed sequence tag; S: Switch region; CSR: Class switch recombination; CDR: Complementarity-determining region; N: Non-templated; P: Palindromic.

## Competing interests

The authors have no relevant conflict of interests.

## Authors’ contributions

YS, ZL, LR, ZW, PW, NL and YZ. All authors participated in concept design, data collection and analysis, drafting and revising the manuscript. All authors read and approved the final manuscript.
